# Supplementary material for: Molecular Cloning and Analysis of an Acetyl-CoA C-acetyltransferase Gene (EkAACT) from Euphorbia kansui Liou
Source: Plants (Basel). 2022 Jun 9;11(12):1539. doi: 10.3390/plants11121539 (PMC9229008; doi:10.3390/plants11121539)
Supplement: Supplementary file 1 [file plants-11-01539-s001.zip › plants-1722325-supplementary.pdf]

Table S1. Primers used in this study.

Primer name	Primer sequence (5'-3')
Primers for subcellular localization	
EkAACT -F1	ATCGGGTACCATGGCCCCAGCTGCAGTC
EkAACT -R1	ATCGGAGCTCAACGCAAACAAGGAAATACTTTCTC
GFP-F1	ATCGGAGCTCATGGTGAGCAAGGGCGAGGA
GFP-R1	ATCGGAGCTCTTACTTGTACAGCTCGTCCA
Primers for transgenic overexpression in *Arabidopsis*	
EkAACT -OE-F	ATCGGTCGACATGGCCCCAGCTGCAGTC
EkAACT -OE-R	ATCGGAGCTCCTAAACGCAAACAAGGAAATACTTTCTC
Primers for real-time quantitative PCR in *Arabidopsis thaliana*	
AtAACT-F	CGTCGCTGATTCACCAACAACAAC
AtAACT-R	CGCCGAATGGAGAGAGCTGAAAG
AtMDC-F	GTGATGGTGACGGCGCAGAC
AtMDC-R	ACGGTGGTGAGAGTACAGAGGTG
AtMK-F	TCAGTGTTCAACGCCGTGGATTC
AtMK-R	GACAGGAGCAGACCTTGGTTCATC
AtHMGR-F	CTGGATCGAAGGACGTGGTAAGC
AtHMGR-R	AGCCTCAACGCTAGTCTTCAACAC
AtHMGS-F	TGGACGTTATGGCCTCGTCA
AtHMGS-R	GCCATGTGGCTTGCTCTCAA
AtSOD-F	ATGAGAAGTTCTATGAAGAG
AtSOD-R	GTCTTTATGTAATCTGGT
AtPOD-F	TCCGGGAGCCACACCATTGG
AtPOD-R	TGGTCGGAATTCAACAG
Atactin-F	GGTAACATTGTGCTCAGTGGTGG
Atactin-R	AACGACCTTAATCTTCATGCTGC

Primers were designed according to the sequence of unigene in transcriptome of *Euphorbia kansui* “ F ” and “ R ” denote “ forward ” and “ reverse ” primers, respectively. “__” denote restriction enzyme cutting site.
